# Road traffic injuries to children during the school commute in Hyderabad, India: cross-sectional survey

**DOI:** 10.1136/injuryprev-2015-041854

**Published:** 2015-12-23

**Authors:** Shailaja Tetali, P Edwards, G V S Murthy, I Roberts

**Affiliations:** 1 Indian Institute of Public Health, Hyderabad, India; 2 Department of Population Health, London School of Hygiene and Tropical Medicine, London, UK

## Abstract

**Background:**

India is motorising rapidly. With increasing motorisation, road traffic injuries are predicted to increase. A third of a billion children travel to school every day in India, but little is known about children's safety during the school commute. We investigated road traffic injury to children during school journeys.

**Methods:**

We conducted a cross-sectional survey in Hyderabad using a two-stage stratified cluster sampling design. We used school travel questionnaires to record any road injury in the past 12 months that resulted in at least 1 day of school missed or required treatment by a doctor or nurse. We estimated the prevalence of road injury by usual mode of travel and distance to school.

**Results:**

The total sample was 5842 children, of whom 5789 (99.1%) children answered the question on road injury. The overall prevalence of self-reported road injury in the last 12 months during school journeys was 17% (95% CI 12.9% to 21.7%). A higher proportion of boys (25%) reported a road injury than girls (11%). There was a strong association between road injury, travel mode and distance to school. Children who cycled to school were more likely to be injured compared with children who walked (OR 1.5; 95% CI 1.2 to 2.0). Travel by school bus was safer than walking (OR 0.5; 95% CI 0.3 to 0.9).

**Conclusions:**

A sixth of the children reported a road traffic injury in the past 12 months during school journeys in Hyderabad. Injury prevention interventions should focus on making walking and cycling safer for children.

## Introduction

### Background

Road traffic injury is a growing public health problem among adults and children in India. In 2013, the rate of road traffic crashes, injuries and deaths per 100 000 population in India was 39, 40 and 11, respectively.^1^ The number of registered motor vehicles in India is increasing by 12% each year^2^ and is projected to increase from 112 million in 2010 to 500–600 million by 2014.^3^ The increasing motorisation is likely to have huge implications for air quality, road traffic injuries and physical activity. Road traffic deaths are predicted to more than double by 2020.^4^

Hyderabad is one of the fastest growing urban areas in India.^5^ Nearly 1 in 14 people report a non-fatal road injury annually, requiring a recovery period of over 7 days. Disability due to road injury in Hyderabad is estimated to be 35 per 100 000 people.^6^ The annual rate of overall road injury among children in 2009 was 11% for boys and 6% for girls,^7^ yet little is known about children's injury during the school commute in Hyderabad.

A third of a billion children travel to school every day in India. Children's travel to school is a routine and necessary activity. But we do not know about the safety of children who walk, cycle or use motorised modes. It is important to identify risk factors because the school trip is a part of children's daily activity and is amenable to interventions.^8^ The objective of this study was to investigate the safety of school journeys in Hyderabad, by mode of travel and distance to school.

## Methods

### Study design

We conducted a cross-sectional survey using a two-stage stratified cluster sampling design. The strata were geographical (mandals, equivalent to boroughs) and administrative (type of school management).

### Study setting

The study was conducted in all 16 mandals of Hyderabad district and 1 mandal of the neighbouring Rangareddy district in 2014. The three main types of school management were included: government, semiprivate and private. Government schools are run by the Central or State Government, semiprivate schools receive a grant from the government and private schools are fully paid for by the parents’ fees.^9^

### Participants

We surveyed children aged 11–14 years, as this is typically an age when children may be expected to travel independently.^10^ In school terminology, it refers to children in grades 6–9. We first randomly selected a school from a list of schools with grades 6–9 in each stratum. Next, the school principal randomly selected sections (ie, classrooms that normally have 30–40 children) in grades 6–9. All children who were present on the day of the survey were included in the study.

### Data collection

We used a validated, self-completion questionnaire with 21 questions for information on various aspects of travel to school. The questionnaire underwent thorough piloting and revision, after two focus groups, seven cognitive interviews and two reliability studies.^11^ This was done to ensure the suitability of the questions for the target age (11–14 years) and to assess the acceptability of the wording, as well as the sequence of the questions.

Detailed instructions were given to children on every question. The question on road traffic injury was ‘During the past 12 months, were you injured in a road incident Road ‘incident’ was defined as ‘any non-fatal injury sustained in the previous 12 months, on the road while going to, or coming from school, due to a collision with another vehicle, or due to a fall or skid from a bicycle or two-wheeler, while standing or walking on the road’. The number of injuries sustained was not required. Children were asked to only report injuries which led to the child missing at least one full day of their usual activities or which required treatment by a doctor or a nurse. This was included to focus only on the more severe injuries.

We used English questionnaires in private schools and a Telugu version (which is the local language of instruction) in government and semiprivate schools. The questionnaire was administered using pencil-and-paper methods during a regular class period and could be completed in 15–20 min. Research assistants with survey and interview experience conducted the survey in the schools, in the presence of the class teachers. They read out each question, allowing plenty of time for marking the responses. The study investigator made monitoring visits to schools to ensure that all questions were read out and explained to the children.

#### Variables

*Outcome*: any road traffic injury on the way to or from school in the past 12 months that resulted in at least one day of school missed or required treatment by a doctor or nurse.

*Exposures*: usual mode of travel; Distance to school. Mode of travel was categorised as *walking, cycling, autorickshaw*, *cycle rickshaw* (commercial three-wheeled passenger vehicles), *school bus* (private), van (private), *RTC bus* (public road transport corporation bus), *motorised two-wheeler* (motorbike), *car* or *train*. We combined *school bus* and *van* because both are private modes providing a door-to-door service. We used Google Earth to estimate distance from home to school, using the school location and the nearest landmark to home reported by children.^11^ We created a categorical variable for distance (<1, 1–2, 2–3, 3–5 and >5 km) to investigate any non-linear relationship with injury.

*Confounding variables*: age, sex, parental permissions for independent travel and type of school. We considered the type of school to be a marker of socioeconomic status and parental influence: generally, government schools in Hyderabad cater to lower income families, semiprivate schools cater to middle income families and private schools cater to higher income families.

#### Study size

We estimated that a sample of 6000 children would be sufficient to detect important differences in the prevalence of road injury by travel mode and distance to school, while allowing for clustering of injury within mandals.

### Statistical methods

We estimated the prevalence of self-reported road traffic injury in the last 12 months during school journeys by mode of travel and distance to school. We used logistic regression to estimate the RR (ORs with 95% CIs) of road injury for each mode of travel adjusting for potential confounding variables. We used the ‘survey’ commands in Stata to account for stratification, clustering and unequal probability of selection, and the ‘test’ command to test the associations in the logistic regression models. We retained variables that remained statistically significant at the 5% level in the ‘best fit’ model. We conducted a sensitivity analysis by fitting the model with distance as a categorical variable. Children who answered ‘*other*’ to the question on their usual mode of travel to school were excluded from the analysis. We analysed data using STATA/SE V.12.0 (Stata, Texas, USA).

The Hyderabad District Education Office permitted the study to be conducted. The ethics committee approved consent being taken from the school principals. The parents/guardians of the children were made aware of the study. We obtained ethics committee approval from the London School of Hygiene and Tropical Medicine, London, UK, and the Indian Institute of Public Health, Hyderabad, India.

## Results

### Participants

Of the 48 eligible schools that were selected, 45 agreed to participate. Three schools refused due to time constraints. Approximately 3% of eligible children in the participating schools were absent on the day of the survey. The total sample was 5842 children, of whom 5789 (99.1%) children answered the question on road injury.

### Descriptive data

The average age of children in the sample was 13 years (SD 1.3 years), with a higher proportion of girls (54%). Of the children who completed the questionnaires, 40 (0.68%) did not provide information on their mode of travel to school. Almost all children (98.7%) provided a valid home address or nearest landmark for the estimation of distance to school.

### Main results

The overall prevalence of self-reported road traffic injury in the last 12 months during school journeys in Hyderabad was 17% (95% CI 12.9% to 21.7%). A higher proportion of boys (25%; 95% CI 19.5% to 30.5%) reported road injury than girls (11%; 95% CI 6.8% to 16.1%).

The prevalence of road injury varied with mode and distance to school (table 1). Cyclists reported the highest prevalence of road injury (33%), followed by children who travel by motorised two-wheelers (20%) and children who walk to school (17%). The lowest prevalence was reported by children who travel by school bus (8%). The prevalence of road injury was highest (25%) among children who travel 2–3 km to school and lowest (9%) among children who travel over 5 km. The prevalence of road injury to children who walked or cycled increased with distance.

Table 2 shows the RRs and 95% CIs associated with travel mode. Children who travelled by bicycle were more likely to report an injury compared with children who walked (OR 1.5; 95% CI 1.2 to 2.0). Children who used the school bus were less likely to report an injury than those who walked (OR 0.5; 95% CI 0.3 to 0.9). This was after controlling for gender, school type, grade and mandal (table 2).

**Table 1:** The prevalence of self-reported road traffic injury by mode and distance to school in Hyderabad

Mode	Prevalence (%)	Distance to school					
	Children in sample (n)	<1 km	1–2 km	2–3 km	3–5 km	>5 km	Total
Walk	%	13	19	30	26	42	17
	n	1859	1330	224	24	8	3445
Bicycle	%	33	30	33	49	0	33
	n	103	108	80	32	1	324
School bus	%	39	4	4	12	4	8
	n	13	31	64	92	207	407
Car	%	54	16	25	4	10	16
	n	16	24	22	40	58	160
Two-wheeler	%	14	17	34	21	4	20
	n	111	146	117	55	25	454
RTC bus	%	4	6	10	22	19	15
	n	37	73	132	140	139	521
Autorickshaw	%	17	7	26	9	11	13
	n	33	93	73	67	104	370
Other modes*	%	62	4	0	16	0	16
	n	9	11	4	12	9	45
All modes	%	16	18	25	16	9	17
	n	2181	1816	716	462	551	5726

*Cycle rickshaw, train and other.

RTC, road transport corporation.

**Table 2:** Association between road traffic injury and travel mode (walking as reference mode)

Mode		OR (95% CI)	
	Children in sample	Model fitted with distance as linear term	Model fitted with categories of distance
Walk (reference category)	3494	1.0	1.0
Bicycle	329	1.5 (1.2 to 2.0)	1.4 (1.1 to 1.9)
School bus	410	0.5 (0.3 to 0.9)	0.5 (0.2 to 0.9)
Car	161	1.3 (0.7 to 2.4)	1.2 (0.7 to 2.3)
Two-wheeler	458	1.3 (0.8 to 1.9)	1.2 (0.9 to 1.7)
RTC bus	531	0.8 (0.6 to 1.2)	0.8 (0.6 to 1.1)
Autorickshaw	374	1.0 (0.5 to 1.8)	0.9 (0.5 to 1.7)
Total	5757		
Test for homogeneity		p<0.001	p<0.001

Logistic regression model including terms for gender, school type, grade and mandal.

RTC, road transport corporation.

Girls were one third as likely to report an injury as boys (OR 0.3; 95% CI 0.2 to 0.4). We found no evidence for associations between road injury and grade, school type, independent travel, perception of safety or physical activity levels. We found that the results of the sensitivity analyses did not differ when categories of distance were used.

## Discussion

### Main findings

This study estimated the prevalence of road traffic injuries during journeys to school in Hyderabad, by mode of travel and distance to school. The principal findings suggest that cycling to school is more hazardous than walking, while travelling by the school bus is safest.

### Limitations

Our estimates of the prevalence of road injury are based on self-reports, which are susceptible to recall bias. Children may have reported injuries that occurred outside of the 12-month period, or did not occur on the school journey or they may not have reported some injuries at all. The relatively long recall period of 12 months may have led to under-reporting of injury, especially if they were minor injuries.^12^ Recall bias might have also occurred if children using some modes (eg, bicycle) were more likely to remember an injury than children using other modes (eg, school bus). This may have led to differential misclassification of the outcome by mode of travel. But there is no reason to suggest that children's ability to recall might differ by distance to school. The mode of travel in which the child was injured was not asked directly, and it was assumed based on their usual mode of travel. It is possible that the injury occurred because a different (and not usual) mode of travel or route was taken, which is a major limitation of our study.

Our definition of injury was one which resulted in at least a day of school missed or required treatment by a doctor or nurse. Some parents may have taken their child with a minor injury to see a doctor or nurse, while other parents may not. Also, our study did not record the number of injuries, severity of injury or location of injury, which limits interpretation. The severity of injury is unlikely to be the same for different travel modes. Specifically, among bicycle injuries, which were most common, it is likely that the majority did not involve collision with a motor vehicle (which usually causes greater severity of injury and disability). Similarly, the striking vehicle for pedestrian injury in the mixed traffic environment in Hyderabad may have been a bicycle, a motorised two-wheeler or an autorickshaw.^7^ The mechanism of injury, however, was not recorded in any detail.

Children who were absent on the day of the survey were not included in the study. It is possible that they are different from those who were present or that they were absent because of a road injury. However, there were very few absent (<3%). This is similar to other estimates of absenteeism (1%) from South Indian schools.^13^ Children are absent usually due to legitimate reasons, including sickness.^14^ Forty children did not provide their mode of travel, 76 children did not give a valid address and 53 children did not complete the question on road injury. These children were excluded from analysis and this may have biased our results.

Due to the cross-sectional nature of the study, we were not able to investigate causal relationships. For example, it is possible that children changed their travel mode following a road injury. Children who were injured when cycling may have changed to a safer mode of travel, such as the RTC bus. This is perhaps less likely in India, where children who walk or cycle do so because they do not have a choice.^15^

The results may have been confounded by other factors. For example, we do not know if recall of road injuries is associated with age, sex, mode or other factors. We were also unable to account for the extent to which characteristics of the road environment, such as vehicle speeds and volumes differ between the mandals where children commute to school. The survey was conducted in the dry season when injuries may differ compared with other seasons. However, we asked about all road injuries in the last 12 months, which should cover all seasons.

Despite these limitations, there was a good response rate (99%). The sample size of 5842 children was higher than in previous studies (1820 and 2809) on injuries in Hyderabad.^7^,^16^ We used a questionnaire that had been shown to be valid and reliable. It showed ‘substantial agreement’ using the kappa statistic for the question on road injury during reliability testing.^11^ While test–re-test is a good measure of reliability, we were unable to validate self-reports against medical reports of the actual injuries due to financial and time constraints. We estimated distance to school based on children's home address and nearest landmark. Because our method was accurate to within 65 m (−30 to 159 m) of the true distance,^11^ we are reasonably confident in the results of the relationship between distance and prevalence of injury. To our knowledge, this study was the first to examine road traffic injuries among children during school journeys in Hyderabad, which is a vital first step for informing policy.

### Comparisons with other studies

Road injury estimates are inconsistent across studies, and this may reflect differences in the operational definition of road injury or origin-destination of trips (any travel and not necessarily school journeys). We estimated an overall prevalence of road injury during school journeys to be 17%. There were no studies in Hyderabad that particularly reported road injury by mode and distance during school journeys. One study reported the reason for being on the road as ‘*going/coming from school/work*’ for 19% of all road injuries.^7^

Cycling was the most risky travel mode, followed by two-wheeler and walking. Our estimate of road injury as a cyclist (33%) and pedestrian (17%) was higher than that reported by a Palestinian study (11% for cycling and 8% for walking).^17^ This is perhaps because it included the activity context (eg, sport) whereas our definition of road injury was specific to school travel.^18^ Our estimates were lower than those reported by another Indian study on road use by children (46% for cycling and 42% for walking).^7^ This could be because the estimates were from a household survey of all road injury among children aged 5–14 years, irrespective of the destination. Another study from Andhra Pradesh used a 3-year recall period for severe non-fatal injuries and found that of all injured children, 52% were cyclists and 20% were pedestrians.^16^

The overall prevalence of road injury among boys was higher than among girls, which is consistent with the results from other Indian studies.^7^ Boys have a higher exposure to bicycle riding compared with girls, and many of the differences in hospital emergency attendance are thought to stem from different exposure rates.^19^ We could not estimate the risk of bicycle injury for girls because the number of girls (n=5) who cycled was quite small, compared with boys (n=319).

Travel by school bus was safer than walking, but the school bus is a private form of transport, paid for by wealthy parents to collect children at the door step. Not all parents can afford to send their children by school bus. The RTC bus (public transport) has approximately 15 million passengers per day and is used by 72% of the population as the primary mode of transport in Hyderabad. Our results show that it is as safe as the car. Private motorised vehicles were associated with a higher prevalence of road injury (20% for two-wheeler and 16% for car), than the public transport modes, and this has been found elsewhere.^20^

#### Interpretation

We acknowledge the limitations of the cross-sectional design and are cautious about interpreting our estimates of the prevalence of road injury by mode, for the reasons outlined above. The results, however, highlight the safety issues associated with children's journeys to school in urban India and that mode choice may alter injury risk. Robust study designs that can answer similar questions more reliably need to be used.^21^ There is a need for future research to evaluate detailed exposure data on the number, severity and location of road injury near school zones. Measures such as the introduction of affordable school buses will be useful to explore. Children's journeys to school are a daily activity that ought to be pleasant and safe. This can only be achieved by improving the overall road safety in Hyderabad, with a strong emphasis on the construction of pavements and cycle lanes.

#### Generalisability

This study presents children's road traffic injury data in all the mandals of Hyderabad, thereby giving a city-wide estimate and satisfying external validity. We estimate that the 5842 children in the sample represent a population of 322 258 children and believe that our results might be generalisable to other urban school populations in India with comparable road infrastructure and travel behaviour.

**New Zealand to invest $1.1 million to prevent and treat child injuries**

The Accident Compensation Corporation is New Zealand's insurance division. It is about to invest $1.15 million to support a programme intended to reach almost 500 000 children over the next 5 years. The goal is to show them how to identify hazardous situations and to train them to respond appropriately if they are at the scene when someone gets injured. “Giving our kids the confidence to call 111 and provide first aid could be the difference that saves a life or reduces the impact of the injury.” Comment: this programme entails ACC teaming with St John Ambulance.

## Conclusions

A sixth of the children reported a road traffic injury in the past 12 months during school journeys in Hyderabad. Considering that a third of a billion children travel to school in India and a majority of them walk or cycle, this is a public health problem of enormous proportions. To prevent these injuries, interventions should focus on making walking and cycling safer for children.

**What is already known on the subject?**

- India is motorising rapidly: motor vehicle registrations are increasing by 12% each year.
- With increasing motorisation, road traffic injuries are predicted to increase.

**What this study adds?**

- A sixth of children aged 11–14 years reported sustaining a road traffic injury in the past 12 months during school journeys in Hyderabad.
- Children who cycle to school were most likely to report injuries.
- Travel by school bus was safer than walking.

**Colorado gives semiautomatic rifles to school safety officers**

A school district in Colorado intends to provide semiautomatic rifles to school safety officers. Officers who noted that sheriff's deputies had long rifles while they only had handguns prompted the purchase. The school superintendent explained that AR-15 rifles were needed in case they were the first to show up at an active shooter scene. Ironically, the same manufacturers who made the one used in the Sandy Hook shooting make these rifles. Comment: rest easy; the safety officers are obliged to complete an annual 20-hour training course.

**Protect children from dangerous cleaning products**

The Royal Society for the Prevention Accidents (RoSPA) is alerting UK parents to protect their children from dangerous household cleaning products. A magnetic notepad featuring safety advice is to be given to 40 000 families by the Southern Health NHS Foundation Trust. The notepad reminds parents to store cleaning products ‘out of reach, out of sight and in a locked cupboard’. As well, parents are urged to store chemicals in their original containers, never pierce or break laundry capsules or tablets, and always close the lid of any product. Comment: alongside CAPT RoSPA uses child safety week to launch this sort of campaign. We rarely (if ever) see the results of these campaigns evaluated but I suspect if they were it would be disappointing.
